# The Effect of Dopaminergic Medication on Joint Kinematics during Haptic Movements in Individuals with Parkinson's Disease

**DOI:** 10.1155/2017/2358386

**Published:** 2017-04-17

**Authors:** Kuan-yi Li, Pei-yi Chu, Kristen A. Pickett

**Affiliations:** ^1^Department of Occupational Therapy and Graduate Institute of Behavioral Sciences, Chang Gung University, Taoyuan, Taiwan; ^2^Healthy Aging Research Center, Chang Gung University, Taoyuan, Taiwan; ^3^Department of Neurology, Chang Gung Memorial Hospital, Taoyuan, Taiwan; ^4^Tan Tock Seng Hospital Rehabilitation Centre, Singapore; ^5^Occupational Therapy Program, Department of Kinesiology, University of Wisconsin-Madison, Madison, WI, USA

## Abstract

This study examined whether altered joint angular motion during haptic exploration could account for a decline in haptic sensitivity in individuals with PD by analyzing joint position data during haptic exploration of a curved contour. Each participant's hand was passively moved by a robotic arm along the edges of a virtual box (5 cm × 15 cm) with a curved left wall. After each trial, participants indicated whether the contour was curved or straight. Visual, auditory, and tactile cues were occluded, and an electrogoniometer recorded shoulder and elbow joint angles during each trial. The PD group in the OFF state had a higher mean detection threshold (4.67 m^−1^) than the control group (3.06 m^−1^). Individuals with PD in the OFF state also had a significantly greater magnitude of shoulder abduction than those in the ON state (*p* = 0.003) and a smaller magnitude of elbow flexion than those in the ON state or compared to the control group (both *p* < 0.001). These findings suggest that individuals with PD employ joint configurations that may contribute to haptic insensitivity. Dopamine replacement therapy improved joint configurations during haptic exploration in patients with PD, suggesting a role for dopaminergic dysfunction in PD-related haptic insensitivity.

## 1. Introduction

Parkinson's disease (PD) has traditionally been characterized as a movement disorder; however, PD is also associated with perceptual deficits, such as decreased sensitivity of olfaction, touch, proprioception, and haptic sensation [1–5]. These deficits may be present in the early stages of the disease, such that olfactory and somatosensory dysfunction have been proposed as early markers of PD [2, 6, 7].

Haptic perception, also referred to as active touch [8], is the ability to perceive the features of an object (e.g., shape, size, and texture) through active touch. This perception modality is the result of sensory and motor integration combining tactile, proprioceptive, and motor signals. Haptic perception is decreased in patients with mild-to-moderate PD compared to that in healthy individuals [3]. Konczak et al. [3] found that 82% of participants with PD exhibited impaired perception of a virtual contour created by the boundary forces of a robot manipulandum; in this study, the median haptic threshold for the PD group was 343% that of the healthy control group. A follow-up study showed that dopamine replacement therapy (DRT) partially restored haptic sensitivity in patients with PD [9], suggesting that levodopa improves motor as well as perceptual function in these patients.

Haptic exploration and kinesthesia rely on the appropriate processing of proprioceptive information. Given that patients with PD have prominent deficits in detecting and integrating joint position and movement [3, 10, 11], the question arises whether deficits in haptic sensitivity result from deficits in the processing of joint position information or from an inability to properly integrate tactile and proprioceptive signals (i.e., a sensory integration deficit). Since PD is typically characterized by motor deficits including the reduction of movement speed and amplitude [12], assessments that use motor performance to measure proprioceptive acuity are not appropriate for evaluating this research question [13]. Instead, we analyzed joint position during haptic exploration of a curved contour before and after medication in order to determine whether restricted or altered joint motion during haptic exploration could account for decreased haptic sensitivity in PD. We predicted that if impaired haptic acuity was related to a motor deficit, there would be characteristic changes in joint motion between the ON and OFF medication states; in contrast, a lack of altered joint motion behavior would suggest that impaired haptic perception was related to a sensory integration deficit.

## 2. Method

### 2.1. Subjects

Six male patients with mild-to-moderate idiopathic PD (mean age, 66.3 ± 2.8 years; age range, 65–72 years; 4 right-handed patients with initial right-side disease and 2 left-handed with initial left-side disease onset) and 6 age-matched male healthy control subjects who were free of neurological disease and upper limb pathology (mean age, 61.0 ± 10.4 years; age range, 46–72 years; all right-handed) completed the study. Handedness was evaluated using the Edinburgh Handedness Inventory [14]. All participants provided written informed consent prior to study participation. The study was approved by the Institutional Review Board of the University of Minnesota.

Individuals with PD were recruited from the movement disorder outpatient clinic at the University of Minnesota. All 6 patients were diagnosed with late-onset (>40 years of age) idiopathic PD. Prior to testing, all patients underwent a clinical examination in which disease severity was rated using the Unified Parkinson's Disease Rating Scale (UPDRS) [15]. All patients were evaluated once in the OFF medication state (after a minimum 12-hour washout period) and once in the ON medication state (at least 1.5 hours after taking their routine antiparkinsonian medication[s] and obtaining a self-reported optimal response), with both evaluations performed by the same neurologist. Daily doses of medication were standardized using an established formula [16] as follows: 100 mg standard levodopa =125 mg sustained-release levodopa =1.5 mg pramipexole =6 mg ropinirole =10 mg bromocriptine =1 mg pergolide (levodopa equivalent values are shown in Table 1). The inclusion criteria for both patients with PD and healthy control subjects included a Mini-Mental Status Examination (MMSE) score ≥ 24 [17] and no previous diagnoses of peripheral nerve disease or another neurological condition. Demographics and clinical data are provided in Table 1.

### 2.2. Apparatus and Procedure

**Figure 1:**
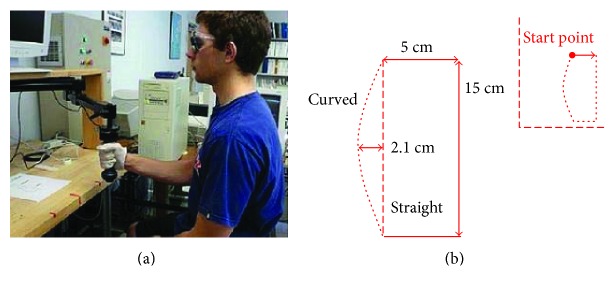
Experimental setup. A robotic manipulandum was used to create a virtual space that was passively explored by the participants. (a) Subjects wore goggles and gloves to occlude visual and tactile cues. (b) The participant's hand was moved along a virtual box (15 cm × 5 cm) that had a left side that was either curved or straight. The maximum curvature experienced by the participant translated to a 2.1 cm deviation from a straight line.

The apparatus and procedure have been described previously [9]. Briefly, participants moved the handle of a 2-joint robotic manipulandum around a predetermined shape bound by a virtual force field (Interactive Motion Technologies, InMotion2). The participant sat facing the robot and holding the handle positioned at the midline of the trunk, just below the shoulder joint. Vision was occluded via opaque glasses, and a synthetic gauze glove was worn to reduce friction between the skin of the hand and the handle to minimize tactile cues (Figure 1(a)). All participants were instructed to gently hold the handle without squeezing it or forcing it in any direction and to allow it to move their hand around the space. Patients with PD used the hand determined to be more affected by PD in the examination by a neurologist, and this hand was matched by healthy control subjects. The participant's hand was passively moved by the manipulandum around the edges of a virtual box (5 cm × 15 cm) with a curved left wall; the curvature of the left side of the box was either convex or straight with curvature values ranging from 0 to 7 m^−1^. A curvature of 7 m^−1^ translated to a 2.1 cm perpendicular deviation from the straight path (Figure 1(b)). Contours were presented at intervals of 0.5 m^−1^, resulting in 15 different curvature values. Using a forced-choice paradigm, participants were required to indicate whether the trajectory was curved or straight at the end of each trial. Data from the perceptual task have previously been reported [9].

Two electrogoniometers (Biometrics Ltd., Cwmfelinfach, UK) were used to measure arm joint angles during the experimental task. A twin axis goniometer was attached across the lateral aspect of the shoulder joint to measure shoulder movement in the sagittal plane (shoulder flexion-extension) and frontal plane (shoulder abduction-adduction). A single-axis goniometer was attached across the elbow joint to measure elbow flexion-extension. The goniometers were connected to a Biopac MP 36 system (Biopac Inc., Goleta, CA, USA), which sampled goniometer data at a frequency of 120 Hz.

The virtual curvature value, perceptual judgment of the participant, and goniometer data were recorded for each trial. Each curvature value was tested 4 times. Healthy participants completed 1 session that included 4 blocks of 15 trials each for a total of 60 trials. Patients with PD completed 2 consecutive sessions for a total of 120 trials; the first session was completed in the OFF state in the morning, and the second session was subsequently completed in the ON state at least 1.5 hours after taking antiparkinsonian medication and achieving an optimal response.

### 2.3. Data Analysis

The joint angles measured were shoulder flexion (ShoFlex) and elbow flexion (ElbFlex) in the sagittal plane and shoulder abduction (ShoAbd) in the frontal plane. Shoulder angles (ShoFlex, ShoAbd) were calculated as the angle between the vector joining the ipsilateral acromion to the lateral epicondyle and the vector from the acromion process towards the hip in the sagittal plane. Shoulder flexion (ShoFlex) and shoulder abduction (ShoAbd) angles were calculated from the same vectors in 2 different projected planes; vectors were projected onto the sagittal plane to calculate the angle of ShoFlex and onto the frontal plane to calculate the angle of ShoAbd. The elbow angle (ElbFlex) was calculated as the angle between the vector from the ipsilateral acromion to the lateral epicondyle and a vector defined by the lateral epicondyle and styloid process of the ulna. Goniometer data were filtered offline with a 4th order low-pass Butterworth filter with a cutoff frequency of 1 Hz. Subsequently, time series data for each trial were cropped and the portion containing curvature exploration was analyzed. A descriptive analysis was performed on all 3 joint angle variables for each participant. Pearson's correlation analyses were used to examine the relationship between joint position and curvature values in all groups and conditions. A multivariate analysis of variance (MANOVA) was used to examine differences in joint angles between groups. Steiger's *Z*-test and Fisher's *Z* transformation were used to examine differences in correlation coefficients within groups and between groups, respectively [18, 19]. The significance level was set to *p* < 0.05.

As presented previously [9], a psychometric sensitivity function was calculated for each individual and group based on participant responses. The detection threshold was defined as the curvature value at the 75% correct response level. The differences in thresholds between the PD and control groups were analyzed using a one-way analysis of variance. Pearson's correlation analyses were used to examine the effects of medication dose and disease duration on haptic sensitivity. Detailed descriptions of the analysis and results for haptic sensitivity have been previously published [9]. Findings from this component of the analysis are presented only for comparison to the new data.

## 3. Results

### 3.1. Typical Joint Configuration during Haptic Exploration

**Figure 2:**
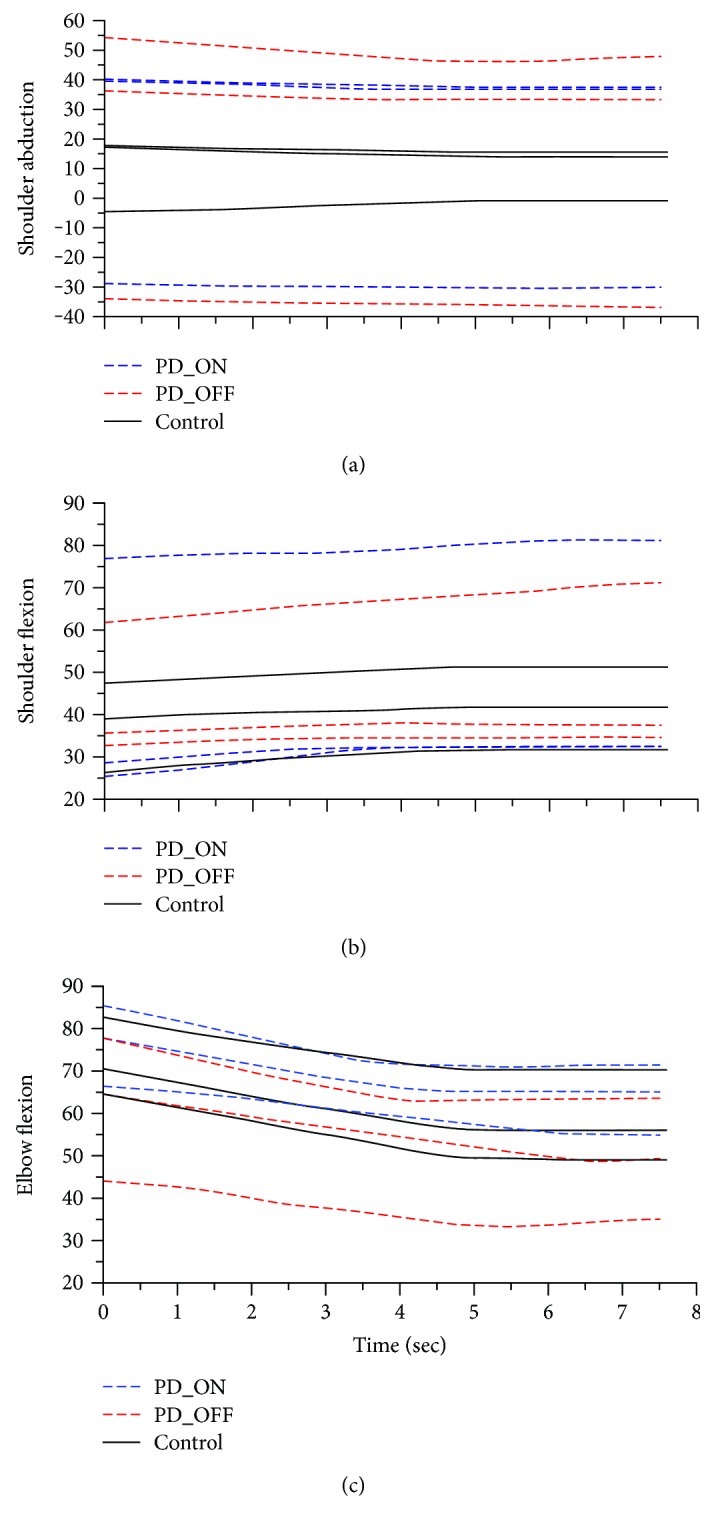
Representative shoulder abduction (a), shoulder flexion (b), and elbow flexion (c) angles derived from 3 patients in the PD group in the ON and OFF states and 3 control participants.

Individuals in both groups used shoulder flexion and elbow extension to explore curvatures. Participants exhibited variable temporal interjoint coordination patterns for shoulder movement in the frontal plane. Some participants started with shoulder abduction, moved towards adduction, and stopped in an abducted shoulder position. Others showed an opposite pattern beginning with shoulder adduction, moving towards abduction, and stopping in an adducted shoulder position. Due to the heterogeneity of the data, no clear pattern could be established.  Figure 2 shows a representative joint angle configuration derived from 3 subjects in each group/condition.

### 3.2. Comparison of Joint Angles

Mean joint angles were calculated within each group for each curvature. There were no significant differences in the 15 curvature values for each joint (*p* = 0.085) within each group. Accordingly, data were collapsed across curvatures for each of the 3 joints (ElbFlex, ShoAbd, and ShoFlex) in each group.  Table 2 presents the range and magnitudes of the 3 joint angles across 15 curvatures (60 trials in total for each participant) for each group. The elbow joint had the greatest magnitude in all 3 groups. There was a statistically significant between-group difference in joint angle (*F*_6,532_ = 7.12, *p* < 0.001). A post hoc analysis revealed that patients with PD had significantly greater magnitudes of ShoAbd in the OFF state compared to the ON state (*p* = 0.003) and smaller magnitudes of ElbFlex in the OFF state compared to the ON state (*p* < 0.001). Magnitudes of ShoFlex were significantly greater in patients with PD in the ON state compared to those in the control subjects (*p* = 0.02).

### 3.3. Group Detection Thresholds (Previously Reported)

The mean threshold was 3.06 m^−1^ in the control group, 4.67 m^−1^ in patients with PD in the OFF state, and 4.04 m^−1^ in patients with PD in the ON state. There was no significant correlation between medication dose and detection threshold (PD-ON: *r* = −0.40; PD-OFF: *r* = 0.32) and between disease duration and detection threshold (PD-ON: *r* = 0.33; PD-OFF: *r* = 0.11). A detailed description of the data analysis and results has been published previously [9].

### 3.4. Relationship between Joint Configuration and Haptic Sensation

In order to examine the relationship between joint angles and deviation from the straight line (curvature), we transformed curvature values into perpendicular deviations from the straight line in a time series and calculated Pearson's correlation coefficients; rShoAbd, rShoFlex, and rElbFlex represent the correlation coefficients for deviation from the straight line and the magnitude of each joint angle, respectively. The mean correlation coefficients for each group are presented in Table 3. Correlation coefficients were all negative values except for ShoFlex, indicating that participants generally increased shoulder flexion, shoulder adduction, and elbow extension to explore the curvature. Joint angles and curvature values for the haptic exploration task were modestly correlated (*r* = –0.33  to  0.28). For all 3 groups, rElbFlex had the highest correlation coefficients and rShoAbd had the lowest correlation coefficients among the 3 joint angles. Results from Fisher's *Z*-test and Steiger's *Z*-test did not reveal statistically significant differences in correlation coefficients between groups; however, results from Steiger's *Z*-test indicated that rShoAbd, rShoFlex, and rElbFlex were all statistically different from one to the other within each group (all *p* < 0.05).

## 4. Discussion

The main purpose of this study was to examine whether restricted or altered joint motion could account for impaired haptic sensitivity during contour exploration in patients with PD. We found that the PD group in the ON state generally used the same joint configurations and had similar levels of haptic sensitivity compared to the healthy control group; however, individuals with PD in the OFF state exhibited differences in shoulder flexion compared to the those of the healthy control group and differences in shoulder flexion and elbow flexion compared to those in the ON state. The correlation coefficient for curvature and shoulder flexion was increased, and haptic sensitivity was improved in patients with PD after the administration of antiparkinsonian medication. Therefore, we suggest that altered joint positioning at least partially accounts for decreased haptic sensitivity in patients with PD.

Previous studies have reported that the central nervous system (CNS) is more accurate in processing end-point control variables than joint excursion variables for kinesthetic-related movements [20, 21]. Mechanoreceptors relay information related to kinesthesia to the CNS; therefore, appropriate joint configurations should facilitate accurate haptic perception during motor tasks. Yet, it is unclear whether joints differentially contribute to haptic sensation in movements involving multiple joints. Scott and Loeb and Tripp et al. suggested that proximal joint positions primarily contribute to end-point position judgments based on computational models [22, 23]. McCloskey et al. reported that proximal joints had greater kinesthetic sensitivity than distal joints; furthermore, proximal joints were more sensitive to joint position and distal joints were better for determining end-point positions [24]. In our study, participants generally used shoulder flexion and elbow extension during the haptic exploration task. Healthy control subjects showed the smallest shoulder range of motion (ROM) in the sagittal plane and the largest elbow ROM. Our data are therefore consistent with the hypothesis that joints function differentially for the generation of perceptual judgments depending on the requirements of the specific motor task.

Galloway and Koshland argued that most multijoint movements of the upper extremities have a shoulder-centered pattern. That is, the shoulder ROM is typically double that of the elbow. If the shoulder ROM is less than half of the elbow ROM and the hand path is straight, the shoulder-centered pattern switches to an elbow-centered pattern [25]. Our findings revealed an average shoulder ROM of less than half the elbow ROM in the control and PD-ON groups; however, the hand path was curved during haptic exploration. We therefore suggest that the task in this study produced a shoulder-centered movement pattern; that is, shoulder acceleration was mainly due to shoulder muscle torque, whereas elbow and wrist acceleration were determined by a combination of muscle torque and significant interaction torque for haptic exploration. Accordingly, the shoulder joint was likely a major contributor to haptic sensation in healthy control subjects and in the PD-ON group.

Previous research has indicated that the CNS uses kinesthetic information from the biarticular muscles during multijoint movements; however, the biarticular muscles generally provide more ambiguous information about limb position and movement than that of the monoarticular muscles [26]. Therefore, the CNS may rely on inputs from the monoarticular muscles as well as cutaneous receptors and joint receptors to resolve ambiguities in kinesthetic information from the biarticular muscles. At the same time, the CNS derives more information from the extent of muscle stretch during overall movement than from any single joint [26]. Taken together, it can be argued that the CNS uses internal representations to integrate sensory inputs for the formation of haptic perception [27].

The current findings are consistent with previous research indicating impaired multijoint coordination and the decomposition of movement in patients with PD [28]. Instead of integrating kinesthetic inputs from the shoulder and elbow joints, patients with PD tend to rely on sensory inputs from a single joint and lack the ability to weight sensory information from different joints to judge limb position and movement [26]. We also found that patients with PD in the OFF state mainly relied on sensory inputs from a single joint during haptic exploration, as there was a larger correlation coefficient for the relationship between elbow flexion and curvature than between shoulder flexion and curvature. In contrast, the healthy control group utilized information from both the shoulder and elbow joints. Therefore, abnormal joint configurations might underlie to impaired haptic sensitivity in PD.

A neuroimaging study of sensory-evoked brain activation after application of a vibratory stimulus to the right index finger found that patients with PD had insufficient activation in several brain regions contralateral to the side of vibratory stimulus presentation and compensatory hyperactivation on the side ipsilateral to stimulus presentation compared to healthy control subjects [29]. The haptic exploration of curvature is based on somatosensory information about the motions and forces experienced during movement. This information is primarily derived from proprioceptive receptors, cutaneous receptors, and mechanoreceptors. A previous study concluded that the perception of actual hand path is mainly derived from proprioceptive feedback rather than force feedback [30], suggesting that haptic perception is generated from the integration of proprioceptive information across several joints and the integration of proprioceptive and tactile information from the fingers and palm. To this end, several studies have provided strong evidence that dysfunction of the cerebro-basal-ganglia loop affects the ability to detect arm position and movement [5, 31–34]. In support of this idea, evidence from animal studies has demonstrated that the depletion of striatal dopamine leads to the inability to use sensory information for motor performance [35]. Other animal studies have shown that basal ganglia neurons, mainly located in the internal globus pallidus, have receptive fields for proprioceptive signals for the processing of both passive and active joint motions [36–38]. In a single-cell recording study of patients with PD undergoing neurosurgery, it was reported that 1/3 of neurons in the nucleus subthalamicus responded to both passive and active movements [39]. Therefore, we speculate that basal ganglia dysfunction was primarily responsible for higher haptic thresholds observed in patients with PD in the OFF state [40]. Moreover, it is possible that DRT produced compensatory changes in PD-related basal ganglia deficits to improve joint and directional specificity. We thus postulate that DRT enhanced neurotransmission in the thalamo-cortico-basal ganglia loop [41] to elicit changes in joint configuration during haptic exploration, resulting in similar patterns of joint angles and lowered haptic thresholds in the PD-ON group.

Previous studies have reported that DRT may actually cause the deterioration of proprioceptive function in PD [11, 42] by producing deficits in proprioceptive processing. Detection thresholds were not statistically different between the PD-ON and PD-OFF states in the current study; therefore, our results suggest that DRT did not have a detrimental effect on proprioception. Inconsistencies between our study and previous studies may have been related to different demographic characteristics of the included PD cohorts. For example, O'Suilleabhain et al. [11] recruited individuals with PD who were approximately 40 years of age, which suggests that patients had early-onset PD and/or an atypical presentation of PD. In contrast, our study examined patients ages of 50–65 years with late-onset PD. Furthermore, different studies have used different experimental protocols for testing in the ON and OFF medication states. O'Suilleabhain et al. [11] retested patients 1 hour after medication, Mongeon et al. [42] retested patients 1-2 hours after medication, and we retested patients 1.5 hours after medication with the additional requirement of a self-reported optimal response. Although all studies administered the UPDRS after medication to ensure efficacy, different time intervals between administration and testing may underlie inconsistent findings between studies. Finally, previous studies used experimental tasks that required participants to memorize a target or joint position for perceptual judgments, which made it difficult to determine whether observed impairments were related to perception or memory. In the current study, we used a passive motion task to exclude the potential influence of motor and memory impairments on perceptual judgments. Thus, the current findings provide an important and unique insight into haptic insensitivity in patients with PD.

## 5. Limitations

The present study had several limitations. First, the power of our statistical analyses was limited by a small sample size. Second, all participants were male, which may limit the generalizability of our findings. Although there is no clear evidence that sex has an effect on haptic sensitivity or joint configuration, potential sex differences should be examined in future studies. Finally, the experimental protocol did not include any functional tasks to directly correlate activity performance with haptic sensitivity. This further limits the generalizability of the current findings to only the tested task. Future research should incorporate functional tasks to examine the contribution of haptic sensitivity to daily activities.

## 6. Conclusions

The findings of the current study suggest that patients with PD employ abnormal joint configurations that contribute to haptic insensitivity. DRT may enhance the neurotransmission in the thalamo-cortico-basal ganglia loop to improve motor ability in patients with PD, resulting in the use of correct joint configurations during haptic exploration and thus increased haptic sensitivity.

## Figures and Tables

**Table 1:** Clinical characteristics and basic demographics of patients with Parkinson's disease.

*N*	Age	Gender	Handedness^∗^	Disease duration (years)	UPDRS				Levodopa equivalent dose	Medication
					Total score		Motor score			
					OFF	ON	OFF	ON		
1	72	M	R	6	32	29	23	20	267	P
2	66	M	R	6	40	25	27	12	500	L
3	65	M	R	11	61	44	41	24	400	L
4	65	M	L	2	41	27	29	15	1000	L
5	65	M	L	9	29	23	18	12	350	L, P
6	65	M	R	5	46	35	32	21	425	L, P

^∗^Handedness was based on the Edinburgh Handedness Inventory (range from 20 [R, right handed] to −20 [L, left handed]) [14]. L: levodopa; M: male; P: pramipexole; UPDRS: Unified Parkinson's Disease Rating Scale (range from 0 to 192, with higher scores indicating higher disease severity [15]).

**Table 2:** Range and magnitude of joint angles in each group.

Group	Control	PD-ON	PD-OFF	*p*
ShoAbd				
Min	−12.64°	−37.57°	−37.70°	
Max	77.52°	50.68°	71.57°	
ROM	5.34 ± 4.12°^AB^	4.40 ± 2.62°^A^	6.60 ± 5.65°^B^	0.005^∗^
ShoFlex				
Min	16.85°	19.67°	−1.76°	
Max	49.70°	76.41°	67.44°	
ROM	3.81 ± 1.34°^A^	4.36 ± 1.15°^B^	4.02 ± 2.10°^AB^	0.08
ElbFlex				
Min	39.83°	27.39°	6.85°	
Max	89.61°	88.36°	89.5°	
ROM	16.20 ± 2.65°^A^	15.26 ± 3.16°^A^	12.43 ± 5.88°^B^	<0.001^∗^

^∗^ *p* < 0.05.

Note: the same letter indicates nonsignificant effect. A different letter indicates a significant difference. ElbFlex: elbow flexion; PD-OFF: Parkinson's disease in the OFF state; PD-ON: Parkinson's disease in the ON state; ROM: range of motion; ShoAbd: shoulder abduction; ShoFlex: shoulder flexion.

**Table 3:** Correlation coefficients between curvature values and joint angles in each group.

	Correlation coefficient (*r*)		
	Control	PD-ON	PD-OFF
rShoAbd	0.04 ± 0.21	−0.12 ± 0.32	−0.13 ± 0.29
rShoFlex	0.28 ± 0.16	0.27 ± 0.17	0.17 ± 0.23
rElbFlex	−0.30 ± 0.10	−0.29 ± 0.09	−0.33 ± 0.11

Note: there were significant differences (*p* < 0.05) between each correlation coefficient within each group. ElbFlex: elbow flexion; PD-OFF: Parkinson's disease in the OFF state; PD-ON: Parkinson's disease in the ON state; ShoAbd: shoulder abduction; ShoFlex: shoulder flexion.

## References

[1] Diamond S. G., Schneider J. S., Markham C. H. (1987). Oral sensorimotor defects in patients with Parkinson’s disease. *Advances in Neurology*.

[2] Herting B., Schulze S., Reichmann H., Haehner A., Hummel T. (2008). A longitudinal study of olfactory function in patients with idiopathic Parkinson’s disease. *Journal of Neurology*.

[3] Konczak J., Li K. Y., Tuite P. J., Poizner H. (2008). Haptic perception of object curvature in Parkinson’s disease. *PloS One*.

[4] Mesholam R. I., Moberg P. J., Mahr R. N., Doty R. L. (1998). Olfaction in neurodegenerative disease: a meta-analysis of olfactory functioning in Alzheimer’s and Parkinson’s diseases. *Archives of Neurology*.

[5] Zia S., Cody F., O'Boyle D. (2000). Joint position sense is impaired by Parkinson’s disease. *Annals of Neurology*.

[6] Konczak J., Krawczewski K., Tuite P., Maschke M. (2007). The perception of passive motion in Parkinson’s disease. *Journal of Neurology*.

[7] Maschke M., Gomez C. M., Tuite P. J., Konczak J. (2003). Dysfunction of the basal ganglia, but not the cerebellum, impairs kinaesthesia. *Brain*.

[8] James J. G. (1966). *The Senses Considered as Perceptual System*.

[9] Li K. Y., Pickett K., Nestrasil I., Tuite P., Konczak J. (2010). The effect of dopamine replacement therapy on haptic sensitivity in Parkinson’s disease. *Journal of Neurology*.

[10] Adamovich S. V., Berkinblit M. B., Hening W., Sage J., Poizner H. (2001). The interaction of visual and proprioceptive inputs in pointing to actual and remembered targets in Parkinson’s disease. *Neuroscience*.

[11] O'Suilleabhain P., Bullard J., Dewey R. B. (2001). Proprioception in Parkinson’s disease is acutely depressed by dopaminergic medications. *Journal of Neurology, Neurosurgery, and Psychiatry*.

[12] Samii A., Nutt J. G., Ransom B. R. (2004). Parkinson’s disease. *Lancet*.

[13] Li K. Y., Su W. J., Fu H. W., Pickett K. A. (2015). Kinesthetic deficit in children with developmental coordination disorder. *Research in Developmental Disabilities*.

[14] Oldfield R. C. (1971). The assessment and analysis of handedness: the Edinburgh inventory. *Neuropsychologia*.

[15] Fahn S. E., Elton R., Fahn CDM S., Jenner P., Teychenne P. (1987). Members of the UPDRS development committee. *Recent Development in Parkinson's Disease. Volume 2*.

[16] Fahn S. (1999). Parkinson disease, the effect of levodopa, and the ELLDOPA trial. Earlier vs later L-DOPA. *Archives of Neurology*.

[17] Folstein M. F., Folstein S. E., McHugh P. R. (1975). “Mini-mental state”. A practical method for grading the cognitive state of patients for the clinician. *Journal of Psychiatric Research*.

[18] Steiger J. H. (1980). Tests for comparing elements of a correlation matrix. *Psychological Bulletin*.

[19] Fisher R. A. (1915). Frequency distribution of the values of the correlation coefficient in samples from an indefinitely large population. *Biometrika*.

[20] Bevan L., Cordo P., Carlton L., Carlton M. (1994). Proprioceptive coordination of movement sequences: discrimination of joint angle versus angular distance. *Journal of Neurophysiology*.

[21] Fuentes C. T., Bastian A. J. (2010). Where is your arm? Variations in proprioception across space and tasks. *Journal of Neurophysiology*.

[22] Scott S. H., Loeb G. E. (1994). The computation of position sense from spindles in mono- and multiarticular muscles. *The Journal of Neuroscience*.

[23] Tripp B. L., Uhl T. L., Mattacola C. G., Srinivasan C., Shapiro R. (2006). A comparison of individual joint contributions to multijoint position reproduction acuity in overhead-throwing athletes. *Clinical Biomechanics (Bristol, Avon)*.

[24] McCloskey D. I. (1993). Detection and execution of movements. *Psychological Research*.

[25] Galloway J. C., Koshland G. F. (2002). General coordination of shoulder, elbow and wrist dynamics during multijoint arm movements. *Experimental Brain Research*.

[26] Sturnieks D. L., Wright J. R., Fitzpatrick R. C. (2007). Detection of simultaneous movement at two human arm joints. *The Journal of Physiology*.

[27] Schwoebel J., Coslett H. B. (2005). Evidence for multiple, distinct representations of the human body. *Journal of Cognitive Neuroscience*.

[28] Seidler R. D., Alberts J. L., Stelmach G. E. (2001). Multijoint movement control in Parkinson’s disease. *Experimental Brain Research*.

[29] Boecker H., Ceballos-Baumann A., Bartenstein P. (1999). Sensory processing in Parkinson’s and Huntington’s disease: investigations with 3D H(2)(15)O-PET. *Brain*.

[30] Song W., Flanders M., Soechting J. F. (2004). Effect of compliance on haptic perception of curvature. *Somatosensory & Motor Research*.

[31] Keijsers N. L., Admiraal M. A., Cools A. R., Bloem B. R., Gielen C. C. (2005). Differential progression of proprioceptive and visual information processing deficits in Parkinson’s disease. *The European Journal of Neuroscience*.

[32] Khudados E., Cody F. W., O'Boyle D. J. (1999). Proprioceptive regulation of voluntary ankle movements, demonstrated using muscle vibration, is impaired by Parkinson’s disease. *Journal of Neurology, Neurosurgery, and Psychiatry*.

[33] Schneider J. S., Diamond S. G., Markham C. H. (1986). Deficits in orofacial sensorimotor function in Parkinson’s disease. *Annals of Neurology*.

[34] Jobst E. E., Melnick M. E., Byl N. N., Dowling G. A., Aminoff M. J. (1997). Sensory perception in Parkinson disease. *Archives of Neurology*.

[35] Henderson J. M., Watson S., Halliday G. M., Heinemann T., Gerlach M. (2003). Relationships between various behavioural abnormalities and nigrostriatal dopamine depletion in the unilateral 6-OHDA-lesioned rat. *Behavioural Brain Research*.

[36] Crutcher M. D., DeLong M. R. (1984). Single cell studies of the primate putamen. I. Functional organization. *Experimental Brain Research*.

[37] Crutcher M. D., DeLong M. R. (1984). Single cell studies of the primate putamen. II. Relations to direction of movement and pattern of muscular activity. *Experimental Brain Research*.

[38] DeLong M. R., Crutcher M. D., Georgopoulos A. P. (1985). Primate globus pallidus and subthalamic nucleus: functional organization. *Journal of Neurophysiology*.

[39] Rodriguez-Oroz M. C., Rodriguez M., Guridi J. (2001). The subthalamic nucleus in Parkinson’s disease: somatotopic organization and physiological characteristics. *Brain*.

[40] Contreras-Vidal J. L., Gold D. R. (2004). Dynamic estimation of hand position is abnormal in Parkinson’s disease. *Parkinsonism & Related Disorders*.

[41] Clissold B. G., McColl C. D., Reardon K. R., Shiff M., Kempster P. A. (2006). Longitudinal study of the motor response to levodopa in Parkinson’s disease. *Movement Disorders*.

[42] Mongeon D., Blanchet P., Messier J. (2009). Impact of Parkinson’s disease and dopaminergic medication on proprioceptive processing. *Neuroscience*.

